# Developing a Scale of Care Work-Related Quality of Life (CWRQoL) for Long-Term Care Workers in England

**DOI:** 10.3390/ijerph19020945

**Published:** 2022-01-15

**Authors:** Shereen Hussein, Ann-Marie Towers, Sinead Palmer, Nadia Brookes, Barbora Silarova, Petra Mäkelä

**Affiliations:** 1 Department of Health Services Research and Policy, Faculty of Public Health and Policy, London School of Hygiene and Tropical Medicine, London WC1E 7HT, UK; Petra.Makela@lshtm.ac.uk; 2 Centre for Health Services Studies (CHSS), University of Kent, Canterbury CT2 7NF, UK; A.Towers@kent.ac.uk (A.-M.T.); N.K.Brookes@kent.ac.uk (N.B.); 3 Personal Social Services Research Unit (PSSRU), University of Kent, Canterbury CT2 7NF, UK; S.E.R.Palmer@kent.ac.uk (S.P.); B.Silarova@kent.ac.uk (B.S.)

**Keywords:** organisational psychology, wellbeing, social care, COVID-19, scale development, EU, workforce, stress

## Abstract

Background: Long-term care (LTC) workers are subjected to structural and inherent difficult conditions that are likely to impact their quality of life at work; however, no agreed scale measures it. This study aims to develop a scale to measure the work-related quality of life among LTC workers in England (CWRQoL). The study establishes the domains/sub-domains of CWRQoL, investigates the tool’s utility and collates information on existing supporting strategies for CWRQoL. Methods: We adopt a mixed-methods approach employing inductive/deductive processes at three stages: (1) a scoping review of the literature; (2) interviews and focus groups with frontline LTC workers, managers and LTC stakeholders; and (3) a content validity consensus survey. Results: CWRQoL is composed of seven domains (and 23 sub-domains). Additional domains to those in the literature include financial wellbeing, sufficient time for building relations, managing grief and emotions associated with client death and end of life care. Stakeholders identified several benefits and challenges related to the CWRQoL tool’s utility. COVID-19 significantly impacted LTC workers’ mental wellbeing and spillover between work and home. Conclusions: The study highlighted the complex nature of CWRQoL and provided a solid ground for developing and validating a CWRQoL scale.

## 1. Introduction

Long term care (LTC), or social care in England, describes a range of activities to support people who need personal and social care, including older people and adults living with disabilities. In England, LTC roles include social work, personal care and practical support for adults with a physical disability, a learning disability, or physical or mental illness, and support for their carers [1]. The sector has been experiencing many long-standing challenges exacerbated during the COVID-19 pandemic [2]. These challenges include limited access to publicly funded LTC services, a fragmented care market, demographic trends increasing demands and the inability to attract and retain a sufficient supply of care workers [3,4]. It is estimated that in 2020, 1.54 million people worked in LTC care in England, with a vacancy rate of 7.3% and a turnover rate of 30.4% [5]. The workforce comprises a high prevalence of female workers (82%) with an over-representation of workers from ethnic minority groups who usually face worse employment outcomes within the sector [6].

LTC work has long been characterised as ‘low-skilled’ in policy debates in the UK and when discussing the role of migrant workers within the context of Brexit. Yet, recent consultations highlight that LTC workers are required to possess strong interpersonal skills, ethics and values, and technical skills, including basic nursing training [7,8]. A body of literature highlights the emotional nature of LTC work with potential implications on workers’ quality of life [9,10,11]. Beyond the specific sector of LTC, staff quality of life at work has been long recognised in organisational studies as a critical influencer on individuals’ health and work performance and organisational functioning [12,13]. For care staff, their wellbeing is perceived to be further associated with the delivery and quality of care [14,15]. 

Care and nursing staff, such as care workers, support workers, social workers, nurses and occupational therapists, are particularly vulnerable to low levels of quality of life (QoL) at work due to the nature of the work and situational factors, such as unfavourable working conditions. LTC is emotionally taxing [16] and linked to emotional and physical stress and burnout [4,11,17,18,19,20]. Care staff work in various settings, including ‘clients’ homes, residential care settings and the community, such as day centres. How LTC is generally structured and delivered includes increased fragmentation of work and persistently low wages [1,21]. QoL at work is also linked in the literature as one factor contributing to the LTC sector’s high turnover and vacancy rates [22,23].

The quality of life at work of LTC workers is also shaped by how their original motivations align with or diverge from their actual work experience. The literature shows that LTC workers are typically motivated to work in this sector because of altruistic reasons linked to their expectations of certain rewards associated with aspects of human interactions and the feelings associated with helping someone in need [24] For LTC workers, a positive experience of providing care, comprising practical tasks and affective relations, is very important in shaping their overall feelings of reward at work. At the same time, while LTC workers often perceive affective relations as a desirable part of their role, this can be at a cost to the care worker through risks of burnout and low pay, as these motivations to care are sometimes exploited by underfunded systems [12,18]. 

As LTC work is co-constituted between the worker, recipient and potentially family members or other informal carers, LTC workers’ affective labour involves continual negotiations within interactions around practical care tasks [25]. Hence, LTC staff are more likely to suffer from ‘moral distress’ while providing care, especially when the organisational structure offers little decision authority for the individual worker [17]. Moral distress can occur when the worker is aware of a moral problem, acknowledges moral responsibility and makes a moral judgment about the correct action [26]. These situations usually arise when a perceived tension between rights and protection occurs, when there is a discrepancy between the perceived right course of action and workers’ ability to take such a decision or when time and task constrain a workers’ ability to provide what they perceive as the ‘right’ care. These tensions may cause dilemmas for LTC workers and are likely to be manifested in feelings of inability to provide the high-quality care they aspire to do [11,17,18].

QoL at work, as a concept, has been extensively explored in the organisational psychology and management literature. It comprises various affective, behavioural and cognitive components, including positive and negative emotions, competence, integrative functioning and autonomy [19,20,21,22,23,24,25,26,27]. There are multiple scales developed to measure these components, with particular attention in the organisational psychology literature to the ‘affective’ QoL at work, for example, the Warr’s scale of job-related affective wellbeing; the job demand-resources (JDR) and Hobfoll’s conservation of resources (COR) model [28,29,30,31]. 

Despite these developments and attempts to measure QoL at work, there is a lack of validated, applicable and sensitive scales to measure care work-related quality of life (CWRQoL). Such measure would recognise the common aspects of LTC work with other sectors and the specific nature of LTC work, such as the time needed to build relations and the emotionally taxing nature of care work. Most research focusing on examining staff wellbeing in human services, including nursing and care work, either confines wellbeing at work to measures of burnout or to single scale items measuring general job satisfaction or intention to quit [32,33,34]. While the literature recognises the importance of measuring wellbeing at the workplace within the human service sector, there is almost no research attempting to develop holistic wellbeing at work scale that is sensitive to the context of emotional work and specifically LTC work. This makes it difficult to draw generalisable conclusions or accurate comparative or causal analyses suitable for effective policy and practice development.

This knowledge gap is due to several factors, but paramount among them is the complex nature of LTC work itself. This requires a holistic scale that captures both the positives and the negatives of care work with adults and older people within specific organisational and delivery arrangements and structures. For example, previous research shows a high level of job stress among care workers. Yet, the literature indicates that developing a one-to-one relationship with care users improves staff job satisfaction despite this process being identified as emotionally and physically demanding on care staff [32]. On the other hand, social support at work and belonging to trade unions have been shown to have positive implications on improving job satisfaction and reducing stress levels of care workers. Yet, there are few active strategies to ensure these are embedded within the delivery of care [19,35]. Hence, there is a need to detail the specific components of quality of life at work among LTC workers. This will allow the development of tailored, sector-specific measures that can accurately capture the multi-directional effects of providing LTC as a formal job within a context of escalating demand and increased service fragmentation.

This paper reports on findings of a study focused on developing an LTC-specific measure of care work-related quality of life (CWRQoL) scale. The purpose is to provide a detailed understanding of how LTC workers QoL is constructed and complete the first phase of the CWRQoL scale development, namely identifying and validating the content of the scale. More specifically, the objectives of this study are:

RQ1. Identify and content validate key domains and sub-domains necessary to construct a care work-related quality of life tool specific to the LTC workforce in England (CWRQoL).

RQ2. Develop a conceptual framework for CWRQoL.

RQ3. Identify existing potential ‘at work’ supporting mechanisms likely to improve CWRQoL.

RQ4: Investigate the potential benefits and challenges in using the scale in practice.

## 2. Materials and Methods

### 2.1. Overall Study Design

This study was specific to England, providing a case study for the proposed scale. A mixed-methods approach was employed to conceptualise the care work-related quality of life (CWRQoL) among LTC workers in England (those working with adults or older people). We used a deductive, inductive and content validation process. The deductive element focused on reviewing the literature and communicating with critical international experts in this field to identify existing relevant scales and their constructs and items [36]. A deductive/inductive method was employed through qualitative interviews and focus group discussions with LTC workers, managers and relevant stakeholders. The findings from the literature review were used as a springboard for discussion to test their applicability to the context of LTC work in England and identify other constructs and items. We conducted a consensus survey for content validity where all participants and experts involved in the project were invited to prioritise and agree on the main domains and sub-domains. We also invited additional LTC workers to contribute to the consensus survey.

**Figure 1:**
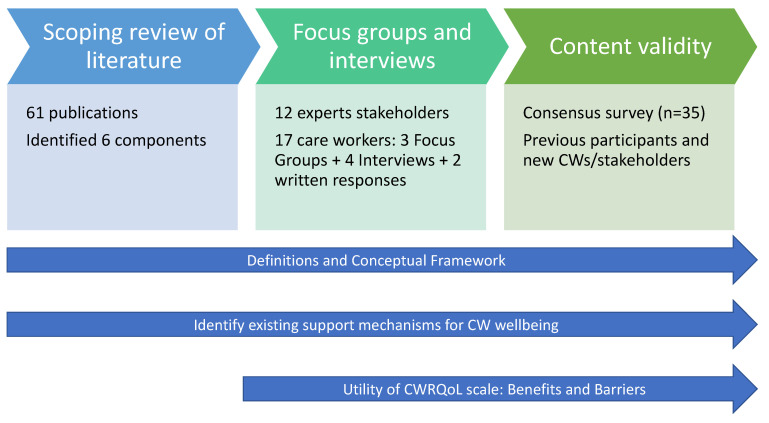
Summary of the Study Design and Participants.

Figure 1 summarises the project design and stages with the numbers of publications identified (and used to inform the discussion guides), and participants contributing to different elements of the research. The core design of developing the CWRQoL scale domains and subdomains (RQ1) consisted of three consequent parts: (1) An inductive process through existing research; (2) An inductive/deductive method gathering direct information from the population group; and (3) Content validity of domains and sub-domains. We used all collated information to develop a working definition for CWRQoL and a conceptual framework of the different domains and sub-domains proposed to construct a new scale specific to care work (RQ2). In addition, we collected specific information from social care experts and stakeholders to understand the utility, benefits and barriers to using a future CWRQoL tool (RQ3); and gathered information on existing in-work support mechanisms for LTC workers during all the stages of the study (RQ4). The fieldwork took place from July to December 2020, and the survey ran for four weeks from June to July 2021. The fieldwork coincided with the onset of the COVID-19 pandemic; we made every effort to focus the discussions on the ‘everyday’ experience of LTC work and added a few questions to collect specific information on the impact of the pandemic.

### 2.2. Data Generation Process

For the inductive Stage 1, we completed a scoping review following a pre-defined study protocol. We developed the search strategy with input from a research librarian from the Academic Liaison Services, Information Services. The searches were conducted between November 2019 and July 2020. To complement the literature review, we compiled a list of 15 international expert researchers in the field of the LTC workforce with interest in work outcomes. We contacted them individually to direct the team to interim findings and ongoing research. After completing the literature and expert review, we started the deductive/inductive stage of work. We employed top-down and bottom-up approaches to engage with different groups within the sector. We planned one-to-one interviews with experts in the field who could reflect on the whole sector, which was complemented by focus group discussions with frontline workers to gain insights on the specifics of daily work.

The fieldwork of this study coincided with the onset of COVID-19 in March 2020, and recruitment during this period was problematic despite exploring various routes and options. Conducting focus group discussions with frontline care workers proved difficult as it was impossible to find an agreeable time for a group to meet virtually. We ran three of the planned six focus group discussions with frontline workers and substituted the rest with individual interviews and written responses. In total, we completed 12 interviews with social care stakeholders between July and September 2020; conducted three focus groups and four interviews, and two written responses with frontline workers, with a total of 17 care workers and managers between July and December 2020. Stakeholders included Chief Executives, Directors, an academic lecturer, a workforce statistician and an independent consultant in social care in England. They were employed by various organisations, including charities, a think tank, workforce and care sector organisations and government. Frontline participants included six care managers, ten frontline staff and one deputy manager/care worker and worked in care homes, community support and home care. 

Stage 2 of the work aimed to explore the meaning of CWRQoL and establish if the components identified in the scoping review related to the actual experiences of frontline care staff and managers and identify any additional or missing components. Additionally, for stakeholders’ interviews, we gathered data on how the sector could use a CWRQoL tool, including the benefits and barriers to having and using the tool, the influence of employers on CWRQoL, and strategies and policies to support CWRQoL.

Stage 3 validated the content of the scale and prioritised domains and sub-domains through a consensus survey. An online survey was developed and distributed to all participants who took part in Stage 2 and a set of targeted new participants and ran for four weeks (June–July 2021). Pre-coded questions were designed to capture the scale domains and sub-domains identified through the study’s earlier stages. We included optional free-text responses for participants to explain or make further suggestions. We also had questions on why improving care workers’ quality of life at work is important. Furthermore, we included questions relating to the impact of COVID-19 on care workers’ quality of life at work and on existing and needed support mechanisms. We drew on the qualitative data from Stage 2 to guide the language and style of the questions and modified these after piloting with three care workers who offered detailed feedback. The pilot phase also highlighted the need to include elements related to dealing with grief related to clients’ death.

The survey included the domains and sub-domains identified at stages 1 and 2; participants were asked to indicate their perceived importance of each of these domains and their specific sub-domains and items. Respondents were asked to select from a 4-point Likert scale ranging from ‘strongly agree’ to ‘strongly disagree’ for each domain, sub-domain and item. This survey design invited respondents to reflect on their views rather than a neutral position. Tailored questions were included for self-employed care workers and those directly employed by a service user. Table 1 summarises those participating in stages 2 and 3 of the study. Due to the in-depth investigative nature of this study, the sample of participants was not intended to be a fully representative sample of all care workers in England. However, Table 1 shows good representation across gender, main client group, care settings (home, residential and live-in care) and job roles. 

### 2.3. Analytical Approach

For the scoping review, a data extraction form was developed. All data were extracted by (BS), 10% checked for accuracy by (NB) and another 10% by (SP) any disagreements were resolved by (SH) and (AMT). The quality of the selected studies was not formally assessed. The study’s key characteristics were summarised, and extracted information was analysed using a narrative approach. Key definitions of Work-Related Quality of Life (WRQoL), components, and measures were tabulated and combined. After a process of familiarisation with the data, each group was qualitatively coded by one reviewer (BS) and discussed with (NB) and (SP). A total of 5979 records were identified and assessed at the title and abstract level. We evaluated 225 full-text articles for eligibility and included 44 studies out of those. We identified 17 other papers through reference lists of eligible full-text articles and our network of experts. In total, we included 61 publications in the qualitative synthesis. Almost half of the studies (n=26) included participants living in continental Europe; 12 studies were conducted in the USA and nine in Canada. Six studies were conducted in the UK or England, three in Australia, two in Israel, one in each of South Africa, Taiwan and Japan. The participants and context varied across studies representing the diversity and complexity of professions and adult social and community health care settings [37]. 

We used a framework approach [36] to organise and code primary data generated through qualitative interviews and focus group discussions. This involved a five-step process of 1- familiarisation, 2- identifying a thematic framework, 3- indexing, 4- charting, and 5- mapping and interpreting the data. All transcripts were uploaded to NVivo (release 1.5) to assist this process. One researcher (SP) read through all the transcripts as part of the familiarisation process, making notes on recurrent themes and critical points. The initial thematic framework was derived by the a priori issues of the research questions and the findings from the scoping review and refined using the notes from the familiarisation stage. The framework continued to develop as critical themes and associations became more prominent during the following stages. The transcripts were indexed by identifying quotes that corresponded to the themes. A second researcher (NB) reviewed 40% of the coded transcripts at this stage to check for agreement of indexing and the attribution of quotes to themes. The charting stage involved arranging the indexed data into charts with themes and subthemes organised by individual cases. This allowed for the participants’ views to be summarised and compared. Finally, the mapping and interpretation stage involved reviewing the charts and notes to look at patterns, connections, and contrasts between participants’ experiences.

We analysed the survey data to identify convergence in opinions. A consensus that an item was of high importance was determined when endorsed as ‘Strongly Agree’ by 40% or more of the respondents. The items were summarised and tabulated (according to domains established in earlier stages) to indicate their relative prioritisation by respondents. Where participants had chosen to elaborate on their views in free text comments, we analysed these qualitative data to help understand the quantitative findings and to identify specific examples in response to items (such as “describe any other forms of support you have received as a care worker”). Data were triangulated and analysed through an iterative process to construct a framework of CWRQoL. The initial framework was discussed with the project advisory group, and feedback was reflected on with further elaboration on the framework and definitions by the research team.

## 3. Results

### 3.1. Identifying the Domain and Sub-Domains Constituting the CWRQoL 

The scoping review [37] indicated an absence of agreement on a definition and measurement of CWRQoL in adult social care. As a result, there is limited evidence on how to improve CWRQoL among people working in adult social care. The scoping review suggested six critical components of WRQoL: (1) organisational characteristics, (2) job characteristics, (3) mental wellbeing, (4) physical wellbeing, (5) spill-over from work to home, and (6) professional identity. The review highlighted a lack of agreement on what WRQoL is, especially in the context of LTC, with no standardised means to measure it in adult social care. Furthermore, the scoping review identified six scales that have been used in the health and social care context. However, the most common components measured by these scales are organisational characteristics, job characteristics and mental wellbeing. None of the identified scales have attempted to measure all the six components of WRQoL. 

The qualitative data analysis identified three broad components of care work that impact care workers’ quality of life. These are: (1) characteristics related to the organisation worked for; (2) features related to the nature of care work and (3) the image and professional identity of care work. Furthermore, the analysis showed that these three broad areas impact the quality of life of care workers in four main dimensions: (1) impacting their physical, (2) mental/emotional and financial wellbeing, and (3) spill-over mechanism from work to home. Table 2 lists key themes and sub-themes presented in the consensus survey that derived from the analysis of the qualitative interviews, focus groups and piloting as shaping CWRQoL.

In terms of care organisations’ characteristics, there were several themes identified through the groups and interviews that could be described as ‘working culture’: communication (with manager and team); working conditions; and working hours (flexibility vs. long shifts); and financial wellbeing (e.g., low pay). Both care workers and managers highlighted the importance of good communication to CWRQoL. One care worker who also had a part-time role as manager appreciated having a manager that was always available and approachable if there was an issue:

*Oh yes, definitely. And she’s on-call twenty-four seven, I mean you know that if you’ve on a night shift, you’ve got a problem at two o’clock in the morning, you just give her a ring. (Interview, care worker/manager, care home, female)*

Opportunities for learning and progression were also essential aspects of the job. Participants emphasised the feeling of meaningful work and conveyed a sense of being invested in, valued and recognised by the organisation. Training also increased their confidence and knowledge when doing their jobs. However, it was primarily felt that these opportunities were lacking:

*Yeah, I would like to progress up the career ladder. But I just feel like there isn’t really much career progression up the ladder. (FG interview, care worker, care home, male)*

Regarding the nature of care work, participants identified factors such as matching the right person to the job, autonomy and control and having time to do the job as necessary for CWRQoL. For example, care workers appreciated when they were given autonomy to make decisions in their daily working if they adhered to general principles and guidelines:

*And in terms of autonomy and the decision making, again my experience of our company likes to have staff taking the initiative, of course always working within the same law and within the guidelines, always following the support plans. Still, the attitude is to always try to have staff which is proactive and they like to have staff taking initiatives and besides we are required to take the decision basically on an everyday or daily basis, and I don’t mind, I like it, I like it. (FG, care worker, community support, male)*

Professional identity and being recognised for doing meaningful work was highlighted as influential in shaping CWRQoL. Such recognition was felt to substitute the challenging working conditions, including poor pay:

*Yeah, well, like MS3 and [MS] said, I think support workers need to be recognised more than the support workers we have. Yes, they are underpaid, and they are the ones doing it because they are ambitious and because they are caring. And some of them—yes, we all need money to live, but at some point, some of them are doing it because it’s rewarding for them. This is how they are here to care for people. And I do think like in an ideal world, they should be recognised more. (FG, manager, community support, male)*

Other areas of work that participants felt to be important to their quality of life at work were having meaningful relationships with clients, having enough time to complete their work to high standards and building necessary relationships with clients. In-depth qualitative data obtained from care stakeholders (top-down) and frontline care workers and managers (bottom-up) confirmed the six dimensions obtained from the inductive process in Stage 1. They identified the financial wellbeing of care workers as an additional component. Poor wages and zero-hour contracts were considered to impact LTC workers’ financial wellbeing in that it affected whether they were able to earn enough money to meet their needs. Furthermore, zero-hour contracts offered a lack of financial stability. It meant that care workers worked overtime and took on additional work with different care organisations to afford to meet needs.

*And because of—it is low paid, and obviously they are trying to get work anywhere—and like [MS2] said, they might take annual leave here and go and work somewhere else. I even know people that will do some shifts during the day and go and do a night shift, just to get the money, because it’s not enough of what they need to live. And yeah, I think it does affect them differently in a way where they just want to earn more and have a better living. (FG, manager, community support, male)*

The consensus survey asked participants to identify the three most important domains derived through the inductive/deductive processes of the study and to indicate the order of importance. The findings are based on how the survey respondents (*n* = 35) agreed or strongly agreed on these priorities impacting CWRQoL. The order of agreements placed financial wellbeing as the most important factor (74%) followed by mental/emotional wellbeing (54%); features of the organisation and the nature of care work came at the third place of priorities (46%); on the fourth place was the spillover impact of work on home life (34%). Table 2 lists the results of content validity of the CWRQoL scale domains and sub-domains. Table 3 presents the process of obtaining the final set of domains, sub-domains, and items through the three stages of this study. The consensus survey included further questions on the consequences of improving CWRQoL; the most strongly endorsed consequence was to ‘improve the overall delivery of care’ (strongly agree: 85%), followed by ‘reducing workers’ stress’ (68.6%); improve care workers’ overall happiness and wellbeing (65.7%) and ‘improve clients’ quality of life’ (65.7%) and the care worker-client relationship (54.3%).

### 3.2. Utility of CWRQoL Scale

One of the aims of the stakeholder interviews undertaken in Stage 2 of this study was to provide further details on the utility of a CWRQoL scale in the LTC sector. Interviewees identified two main ways that a CWRQoL tool could be used. The first was to give leadership insights into staff experience, which could lead to improved relationships between management and staff, and allow the early identification of problems or issues:

*I think it will probably give a better insight for the leaders of those organisations if they really took it seriously. ‘Cos, you know, everybody works really hard in social care, so actually you very rarely take the time to stop and reflect and think about a different set of issues, so there’s a positive there. And it might forge better relationships between leadership and workforce, that might—and, you know, that is much needed, I think. (Stakeholder interviewee 04)*

The second was that a tool could be used in benchmarking across the sector and potentially used as a quality indicator:

*One of the things they will always say, “Oh, but each care home is different. Each care agency is different, so generalising is probably not a good idea, or benchmarking is not a good idea.” But then I think, well, all hospitals are different too, aren’t they? So, if we can do it in the hospital sector, the health sector, why can’t we do something similar for the social care sector as well? (Stakeholder interviewee 11)*

Regarding how comprehensive the coverage would need to be for a tool to be useful, there were differing opinions on whether a local or national focus was most appropriate. There was a role seen for a tool at a local level in monitoring but having national coverage could be beneficial in looking at the impact of national initiatives and policies. Taking a sector-wide view of wellbeing was of increased relevance because of the COVID-19 pandemic.

**Figure 2:**
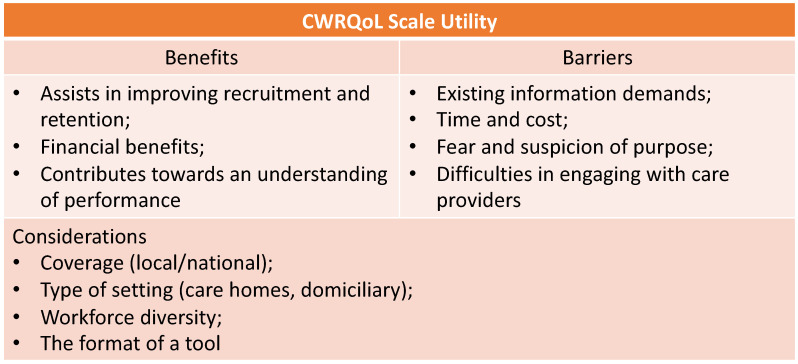
Identified benefits, barriers and considerations of the utility of a CWRQoL scale in the English LTC sector, Stage 2 stakeholders’ interviews.

Stakeholders highlighted some of the potential benefits of the tool in enhancing recruitment and retention in the sector, including financial benefits to care organisations, and in contributing towards an overview of performance. On the other hand, stakeholders identified potential barriers to using a tool in the sector. The latter including existing information demands on care providers, time and cost; as well as some fear and suspicion of the purpose for collecting such data and finding effective ways to engage providers. Furthermore, stakeholders identified several issues in connection with how a CWRQoL tool would fit with care work, including considering the type of setting, workforce diversity and the format of a tool. Figure 2 summarises the benefits, barriers and considerations of the utility of the CWRQoL scale in the English LTC sector.

### 3.3. Strategies to Support CWRQoL

The systematic review for this study highlighted a lack of studies specific to implementing and evaluating strategies to support CWRQoL. The review identified two randomised controlled trials specific to such a strategy. Berendonk et al. [38] tested an intervention (DEMAIN) to improve job satisfaction and reduce work strain among nursing staff in 20 LTC facilities specific to dementia care in Germany. Biederman et al. [39] intervention (TENSE) focused on training interventions on CWRQoL in 18 Dutch dementia specialised care facilities. It concluded that the programme had no significant effect on any CWRQoL components.

The study further collected primary data on existing support mechanisms in England. Existing services and interventions were often a mixture of formal (employer-led) and informal (led by teams or particular managers). Participants indicated that formal, employer-led interventions were minimal and often tertiary in nature, and mostly not bespoke to the nature of the LTC sector. These interventions primarily focused on communication pathways to identify and resolve issues and took one of the following forms:

- Staff surveys and feedback from senior management.
- Employee consultative committees for staff to raise issues with senior/executive management and human resources.
- Employee assistance programmes (confidential helplines, counselling and signposting to further resources and sources of help).
- Financial assistance for staff experiencing hardship during COVID-19.

Some employers also offered a national reward and retention schemes to staff, such as discount schemes (e.g., Perkbox) and cycle to work schemes (discounted bikes).

Secondary interventions designed to support the workforce coping with the daily demands of their caring roles were only mentioned by managers and people in strategic roles as essential to enhancing CWRQoL but were not mentioned by frontline care workers themselves. Given that such interventions often place responsibility for being ‘well’ on individuals (e.g., resilience and coping strategies), perhaps these were not perceived (or valued) by staff as formal interventions for supporting their wellbeing at work. However, informal strategies were frequently mentioned and valued. Some managers went ‘over and above’ to support their teams through small-scale primary interventions such as offering care workers the flexibility of shifts, breaks, and annual leave. Managers also described supporting their teams to cope with work-related stress through informal secondary interventions designed to make staff feel supported and valued, such as phone/zoom calls to ‘check in’ (even when not working themselves); and treats during shifts (e.g., pay for a takeaway once a month).

Although welcomed by staff, such informal interventions were felt to be fragmented and unsustainable. They may even lead to staff in teams with less proactive managers feeling unsupported and resentful. Care workers and managers knew what primary interventions they needed to improve staff wellbeing. These included having more resources, more time to complete care tasks and adequate number of staffing. However, when asked explicitly what would help their wellbeing at work, participants seemed to constrain themselves to strategies employers could do now, within current funding models such as mental health first aiders in each service/team; better holiday allowances; predictable shift patterns; social activities and team building events; and phone lines/zoom links for lone workers/staff feeling isolated or lonely. As part of this study outputs, we produced a guide to support LTC employers to support CWRQoL with recommendations for actions [40].

### 3.4. The Potential Impact of COVID-19 on CWRQoL

The study coincided with the onset of COVID-19 but was designed to capture the components of CWRQoL in usual conditions. Hence, during the fieldwork, the research team emphasised that participants should make every attempt to reflect on ‘normal’ care work activities and discuss any specific implications related to COVID-19 separately at the end of the interviews/focus groups. A similar attempt was made in the wording of the consensus survey with dedicated questions specific to the potential impact of COVID-19 on CWRQoL.

Managers participating in the interviews and focus groups indicated that COVID-19 had heightened the challenges associated with the recruitment of care workers, particularly when it came to recognising the skill sets needed to perform these jobs. These new pressures exacerbate the perception that care can be performed by ‘anyone’ and adversely impact the professional identity and public recognition of care workers, which has been identified as a critical component of CWRQoL.

*Yeah, so, one of the things I’ve been trying to do is call social care workers, social care professionals, ‘cos the language, particularly at the moment ‘cos of COVID’, is about worker, right? That fails to recognise, I think, the kind of amount of skillsets that people actually have and they actually need to do their jobs, and that leads into that poor status and esteem that’s given to that role by society more generally but also, you know, they are a care worker, let’s send a care worker, rather than a social care professional. So, I think there’s an esteem issue that resonates with care workers, “I am just a care worker,” is what you’ll hear a lot. You know, “Nobody listens to me, I am just a care worker.” So, I think there’s an esteem issue that plays on people’s minds a bit and affects people’s quality of life (Stakeholder interview, Dementia charity, male)*

Participants in Stage 2 and 3 of this study highlighted a change in the public perception of care work in England: “Since COVID, there is a greater public recognition of the importance of care work”. However, such recognition was felt by many as superficial with no real change attached to it:

*“The fact that the public came out and ‘clapped for carers’ but then all of the attention went away and the care sector is now in a worse position than ever has been detrimental for many people in the sector and how they view themselves and their work.” (Survey respondent, 79980623)*

*“Although lip service was paid to carers in media (clap for carers etc.) we still feel completely forgotten and disparaged often by government.” (Survey respondent, 79999293)*

The consensus survey asked participants to identify the domains of CWRQoL that were negatively, or very negatively, affected by COVID-19. These were ‘work-life spilling over into home-life’ (76.5%) and ‘mental wellbeing’ (74.3%). When asked about the most helpful support they received during COVID-19 to improve CWRQoL, participants identified ‘COVID financial assistance’ in their free-text responses.

## 4. Discussion

Through the iterative stages in this study, we have identified domains, sub-domains and items, that hold significance for participants in making sense of the concept of CWRQoL. The domains of CWRQoL and their contained sub-domains and items vary, from concrete and objective (such as employer characteristics) to abstract, subjective experiences (such as emotional wellbeing). Through their effect and interactions, these domains formulate care workers’ CWRQoL. Drawing on findings from this study, we propose theoretical links that unite the domains of care workers’ CWRQoL within a dynamic construct; that is, one that varies with time and experience. We thus propose the following definition of CWRQoL:

*At a particular time, a care worker’s work-related quality of life corresponds to their experiences of work tasks and interactions, determined by and rewarded within an employment context in which interacting emotional, physical, social and financial components of wellbeing are impacted in work life and non-work life, and potentially shape their engagement with care.*

This definition integrates concrete aspects of the work while acknowledging the subjectivity of the overall construct and the inseparable relationship between wellbeing at work, wellbeing outside work, and possible impact on the work itself. It has the potential to distinguish between individuals within the same employment context and performing related job roles.

**Figure 3:**
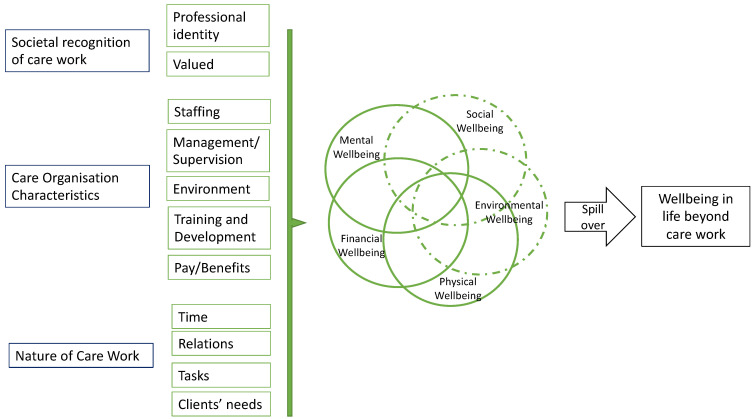
Care Work Related Quality of Life (CWRQoL): a theoretical model.

We expand on this definition through a theoretical model constructed by analysing findings across the three study stages (Figure 3). The hypothetical pathways commence with concrete employment and individual job factors, which are in a relationship with subjective factors of how the care worker experiences the work and its impacts on them, with consequences for wellbeing in their work life, in turn impacting wellbeing in their non-work life. These pathways do not assume positive or negative relationships. Instead, they have the potential to vary between individuals and within individuals’ experiences. The components of the theoretical model are detailed in Table 3 and presented graphically in Figure 3.

The CWRQoL framework suggests three underlying (latent) factors: (1) societal recognition of care work; (2) care organisation characteristics and (3) nature of care work to influence care workers mental, financial, and physical wellbeing, which has an impact on workers’ broader life through the mechanisms of ‘spillover from work to life’ component. The framework also recognises other wellbeing components not fully captured in this study, including social and environmental wellbeing. The latter might have particular importance when care is delivered in peoples’ own homes, such as homecare and live-in care. The proposed CWRQoL framework identifies the ‘environment’ of work as a critical domain related to the care organisation characteristics. It should measure the nature and type of the care settings. However, the manifestation of the workers’ environmental wellbeing is not fully captured and might require further elaboration when the scale is further developed, piloted and validated.

Some existing scales could be adapted to collect some of the domains identified as part of the proposed CWRQoL, especially those related to care organisation characteristics. For example, supervisor and manager support could be measured by (or a modified version of) the Organizational Stress Questionnaire (VOS-D) [41] and measuring tasks could start by adopting the Task and Job Analysis Tool [42], which was further developed for the LTC sector [38]. The findings of the scoping review identified different existing scales that have been used previously in the literature to measure various domains [37].

On the other hand, this study identified several domains and sub-domains that are very specific to the nature of LTC care and the English context. These feature heavily within the nature of care work factor, where time, building relations and the specific clients’ needs are presented. These mainly link back to the existing literature on the emotional nature of LTC work [16] and the main motivations and drivers of many care workers to join the sector in the first place [24]. There are various consequences for care workers’ CWRQoL about the idea that care workers have a sense of ‘calling’ through which they find meaning in their work [43]. Participants who took part in the interviews and focus group discussions as part of this study described a sense of fulfilment from the relationships with clients they acquired through their work. Furthermore, this concept of fulfilment and reward was endorsed by all survey respondents who agreed that ‘feeling a sense of satisfaction from helping others’ was an important factor in care workers’ CWRQoL.

The study highlighted new domains and sub-domains of CWRQoL that were not fully recognised in previous research. Among them is the overall impact on workers’ financial wellbeing, beyond law wages and contract type. While some existing scales attempt to capture some of this, such as the level of wages and job insecurity, these were primarily examined as elements of working conditions [44] with modest, unvalidated, efforts to measure care workers’ perceived financial wellbeing [45]. Financial wellbeing, including how well care workers can manage their finance. has been gaining considerable conceptual recognition [46] but not measured coherently in exiting WRQoL among LTC workers [37]. This study identifies that financial wellbeing includes, but is not limited to, pay, benefits, job (in)security, needing to work in several settings due to low wages and having enough regular income to meet one’s needs. The latter aspect was identified in previous research to be significantly associated with experiencing unresolved stress among LTC workers in England [11].

Another aspect of QoL at work identified by this study is the intersection between building relationships with clients in LTC settings and the impact of dealing with difficult emotions, such as death and grief, on CWRQoL. This was a new component of care workers’ mental wellbeing that has not received been consistently measured in existing scales attempting to measure WRQoL. More broadly, client care needs and building relations were identified as an important domain in shaping the nature of LTC work. Having enough time was an important aspect that influenced CWRQoL and participants recognised and distinguished aspects of ‘time’ to ensure their CWRQoL. For example, participants distinguished between having sufficient time to complete the care tasks, to build relationships with clients, to attend training, to complete administrative tasks and shift patterns. While it was not one of the original objectives of the study, the survey findings highlighted that CWRQoL was perceived to directly impact the quality of care provided and improve clients’ quality of life; such a link has been demonstrated previously in healthcare settings [47].

Interviews with sector experts and stakeholders highlighted the value and benefits of developing and promoting the use of the CWRQoL scale in the LTC sector. The benefits include enhancing recruitment and retention and the financial benefits associated with savings linked to retaining staff and having a standardised tool to assess performance. On the other hand, scaling up the use of such a tool should consider existing information demand, potential time, and cost associated with gathering such information regularly. There were also some reservations in clarifying the purpose of a nationally adopted CWRQoL tool. For example, a standardised CWRQoL scale will be welcomed if it is going to be used to encourage employers to seek support to implement strategies addressing their staff QoL but not if it is used as a performance indicator, which might attach some form of punishment such as reduced quality rating.

The study highlighted a lack of existing supporting mechanisms for CWRQoL, both in previous research and practice. The findings of this study provide much-needed guidance on ways to support LTC workers in general and during the challenging time of the COVID-19 pandemic. COVID-19 has particularly impacted the mental wellbeing of LTC workers and adversely affected the spill-over from work to life. Other research from the UK shows that the pandemic has negatively affected LTC workers’ workload and general wellbeing [48].

## 5. Conclusions

The current study establishes the domains, sub-domains and items of scale specific to measuring the quality of life at work among LTC workers (CWRQoL) and presents the first step of developing a validated scale. The study utilises an inductive–deductive approach to ensure the inclusion of existing research in this area and build on it to establish a detailed and coherent scale that is sensitive to the impact of both the structure and nature of LTC work. We established a definition and a conceptual framework of CWRQoL that integrates concrete aspects of LTC work while acknowledging the subjectivity of the overall construct and the inseparable relationship between quality of life at work, outside work, and possible impact on the work itself. One of the unique contributions of this work is the identification of several context-specific components of CWRQoL such as the importance of time in building relationships and how this interacts with workers’ own quality of life at work. There is a potential for adapting some existing scales to measure more generic organisational characteristics such as staffing, autonomy and managers’ support. However, there is a clear need to integrate an additional set of aspects that are congruent to the emotional nature of work and their implications for the mental, financial and physical wellbeing of care workers. The proposed framework has the potential to distinguish between individuals within the same employment context and those performing related job roles. 

This research is timely where the LTC sector has taken a central stage during the COVID-19 pandemic, where acute workforce challenges are gaining considerable policy and practice attention. The findings of this research will guide the academic development of a coherent tool that will contribute to the broader organisational psychology body of research and draw attention to the importance of improving CWRQoL as an integral part of the sustainability of LTC delivery. The demand for the latter is projected to escalate in the coming decades due to several demographic and social factors, including population ageing and changes in families and communities’ structures.

## Figures and Tables

**Table 1:** Description of participants in Focus Groups and Interviews.

Characteristics	Focus Group Participants (*n* = 11), Focus Group Interviews (*n* = 4), Written Responses (*n* = 2) Total (*n* = 17)	Stakeholder Interviews (*n* = 12)	Consensus Survey (*n* = 35)
Gender	Female, *n* = 8 Male, *n* = 9	Female, *n* = 6 Male, *n* = 6	Female, *n* = 29 Male, *n* = 6
Job Role	Frontline care worker, *n* = 10, Care service manager, *n* = 6 Dual role care worker/manager, *n* = 1	CEO, *n* = 2 Director of Operations, *n* = 1 Statistician, *n* = 1 People Experience Manager Independent Consultant and dignity adviser, *n* = 1 Trustee, *n* = 1 Director, *n* = 1 Director of Clinical Services, *n* = 1 Policy Director, *n* = 1 Project manager for evidence and impact, *n* = 1 Research Director, *n* = 1 Principal Lecturer and Programme Leader, *n* = 1	Direct care role, *n* = 10 Managerial or supervisory role, *n* = 7 Care provider employer role, *n* = 3 Academic or researcher, *n* = 9 Registered professional, *n* = 4 Administrative or Support role, *n* = 2 Policy-maker, *n* = 2 Non-profit or charity based role, *n* = 4 Unemployed, *n* = 1 Other, *n* = 1 (NB survey respondents could identify with more than one role)
Organisation	Care home, *n* = 6 Community support, *n* = 10 Home care, *n* = 1	Workforce organisation, *n* = 2 Government, *n* = 1 Charity, *n* = 4 Think tank. *n* = 1 National Homecare provider, *n* = 1 Telecare organisation, *n* = 1 Home care trade association, *n* = 1 University, *n* = 1	
Years in Current Role	Mean = 3, min = 0, max = 12 Missing, *n* = 6	M = 8, min = 1, max = 15 Missing, *n* = 1	
Age	Mean = 40, min = 20, max = 55 Missing, *n* = 12	-	18–24 years, *n* = 2 25–34 years, *n* = 5 35–44 years, *n* = 5 45–54 years, *n* = 8 55–64 years, *n* = 10 ≥65 years, *n* = 5
Ethnicity	White, *n* = 4 Other ethnic group, *n* = 1 Missing, *n* = 12		
User Group Cared for	Older adults (65+), *n* = 2 Adults of all ages with intellectual and developmental disabilities, *n* = 1 Younger adults (18–64) intellectual and developmental disabilities, *n* = 2 Missing, *n* = 12	-	

**Table 2:** (**a**) CWRQoL scale domains and sub-domains content validity results, Stage 3: consensus survey. (**b**) Sub-domains to be excluded from the CWRQoL according to content validity, Stage 3: consensus survey.

(**a**)		
**Domain and Sub-Domains/Items to be Included in the CWRQoL Scale and their Priorities**	**%**	***n***
**1. Financial wellbeing (74%)**		
Financial wellbeing (enough money to meet your needs)	69	24
Pay and benefits	46	16
Job security	46	16
2. Mental wellbeing * (54%)		
*2.a Burnout/exhaustion*		
Feeling burnt out (unable to cope with work demands)	77	27
Impact of work on mental health (thoughts, feelings, mood)	74	26
Feeling emotionally exhausted at work	74	26
*2.b Satisfaction/motivation*		
Feeling a sense of satisfaction from helping others	71	25
Feeling motivated, enthusiastic or energised by work	69	24
*2.c Affected by loss of clients*		
Impact of grief when a client dies	54	19
**3. Features of the organisation/workplace (46%)**		
*3.a Staffing*		
Sufficient staffing	80	28
*3.b Management and supervision*		
Style of leadership and management	77	27
Feeling supported to do the job	77	27
Supervision arrangements	40	14
*3.c Working environment*		
Feelings of trust and safety within organisation	74	26
Physical work environment	40	14
*3.d Career development*		
Recognition of work achievements	74	26
Availability and access to training	57	20
Opportunities for learning and development	51	18
Having career progression options	40	14
**4. What care workers do in their jobs (46%)**		
*4.a Time*		
Time to appropriately perform care activities	67	24
Time for training	63	22
Working hours and shift pattern	54	19
Time for administrative work (e.g., documenting care)	40	14
*4.b Relations*		
Helping improve others’ quality of life	66	23
Developing relationships with clients	51	18
Feeling responsible for clients	51	18
Feedback from clients/families	51	18
Enabling clients to make their own decisions	40	14
*4.c Role & tasks*		
Clearly defined roles and responsibilities	63	22
Worrying about making mistakes	54	19
A sense of control over own activities	51	18
Variation in your work activities	49	17
Matching staff to the tasks they are good at	49	17
*4.d Care clients’ needs*		
Feeling overwhelmed by needs (e.g., behaviours that challenge)	69	24
Impact of caring for people at the end of life	57	20
5. Impact of work on home-life * (34%)		
Fatigue or other problems that limit what you do outside work	77	27
A positive mood from work that can improve your home-life	60	21
Work-related thoughts that stay with you off duty	60	21
**6. Professional identity as a care worker (26%)**		
*6.1 Feeling valued and respected*		
Feeling respected and valued by your employer	77	27
Feeling respected and valued by colleagues	74	26
Feeling respected and valued by clients	71	25
Feeling respected and valued by other professionals	71	25
Feeling respected and valued by the public	60	21
*6.2 Proud of profession*		
A sense of pride in your profession	63	22
**7. Physical wellbeing (20%)**		
Work-related physical injuries	63	22
Equipment to do the job	51	18
Impact of work on physical wellbeing (e.g., aches and pains)	47	17
Impact of work on healthy behaviours (e.g., eating, sleeping)	47	17
(**b**)		
**Sub-domains to be excluded from the CWRQoL scale: for which <40% respondents ‘Strongly Agree’**	**%**	***n***
**Organisational characteristics**		
Rules and procedures	26	9
**Job characteristics**		
Control over shifts and breaks	26	9
**Spillover from work to home**		
Skills developed at work that can help in home-life	31	11

* Shows the domains for which a negative impact from COVID-19 was most frequently endorsed.

**Table 3:** Development of the CWRQoL domains, sub-domains and items through the different stages of the project.

Stage 1: Inductive (Literature Review)		Stage 2: Deductive/Inductive (Qualitative Interviews)		Stage 3: Content Validity and Order of Importance (Consensus Survey)		
Domains	Sub-Domains	Domains	Sub-Domains	Domains	Sub-Domains	Items
Organisational Characteristics	Working Culture	Care organisation characteristics	Working environment	Features of care organisation (3)	Staffing	Vacancy rate; sufficient staff to client ratio
	Working Climate		Staffing		Management and supervision	Style of leadership and management; Feeling supported to do the job; Supervision arrangements
			Management and supervision		Working environment	Feelings of trust and safety within organisation; Physical work environment
			Diversity and inclusivity		Career development	Recognition of work achievements; Availability and access to training; Opportunities for learning & development; Having career progression options
			Career development			
			Rules and procedures			
Job Characteristics	Job-person match	Nature of LTC work	Time	Nature of LTC work (4)	Time	Time to appropriately perform care activities; Time for training; Working hours and shift pattern; Time for administrative work (e.g., documenting care)
	Autonomy/Control		Relations		Relations	Helping improve others’ quality of life; Developing relationships with clients; Feeling responsible for clients; Feedback from clients/families; Enabling clients to make their own decisions
	Enough time to do the job		Roles and tasks		Roles and Tasks	Clearly defined roles and responsibilities; Worrying about making mistakes; A sense of control over own activities; Variation in your work activities; Matching staff to the tasks they are good at
	Responsibility for people		Care client needs		Care client needs	Feeling overwhelmed by needs (e.g., behaviours that challenge); Impact of caring for people at the end of life
	Learning and Growth		Control over shifts and breaks			
Mental wellbeing and health	Compassion Satisfaction	Mental Wellbeing	Burnout/exhaustion	Mental wellbeing (2)	Burnout/exhaustion	Feeling burnt out (unable to cope with work demands); Impact of work on mental health (thoughts, feelings, mood); Feeling emotionally exhausted at work
	Burnout		Satisfaction and motivations		Satisfaction & motivations	Feeling a sense of satisfaction from helping others; Feeling motivated, enthusiastic or energised by work
	Subjective experience of happiness				Affected by loss of client	Impact of grief when a client dies
Physical Wellbeing and health	Work-related physical injuries	Physical wellbeing	Work-related physical injuries	Physical wellbeing (7)	Work-related physical injuries	
			Equipment to do the job		Equipment to do the job	
			Impact of work on physical wellbeing		Impact of work on physical wellbeing	
			Impact of work on healthy behaviours		Impact of work on healthy behaviours	
Spill-over from work to home	Work related thoughts to stay off duty	Spill-over from work to home	Work related thoughts to stay off duty	Spill-over from work to home (5)	Fatigue or other problems that limit what you do outside work	
			Skills developed at work that can help in home-life		Work related thoughts to stay off duty	
			Fatigue or other problems that limit what you do outside work		A positive mood from work that can improve your home-life	
			A positive mood from work that can improve your home-life			
Professional identity		Professional identity	Valued and respected	Professional identity (6)	Valued and respected	Feeling respected and valued by your employer; Feeling respected and valued by colleagues; Feeling respected and valued by clients; Feeling respected and valued by other professionals; Feeling respected and valued by the public
			Proud of profession		Proud of profession	A sense of pride in your profession
		Financial wellbeing	Enough money to meet needs	Financial wellbeing (1)	Enough money to meet needs	
			Pay and benefits		Pay and benefits	
			Job security		Job security	

Notes: Columns 1–2 list domains and sub-domains identified through the literature review; Columns 3–4 list domains and sub-domains discussed as important by participants in the qualitative interviews and focus groups; Columns 5–7 list domains, sub-domains and items agreed to be essential for CWRQoL. Underlined text indicates newly identified domains and sub-domains at Stage 2 and 3; the numbers on brackets on Column 5 indicate the order of importance of domains as identified during Stage 3.

## Data Availability

The datasets generated and analysed during the current study are not available in any repositories and cannot be shared by the authors because ethical approval for data sharing was not given by the University of Kent Research Ethics Committee and study participants did not give their consent for data sharing. The remaining study materials are available from SH by reasonable request.
